# Effect of Task Specific Exercises, Gait Training, and Visual Biofeedback on Equinovarus Gait among Individuals with Stroke: Randomized Controlled Study

**DOI:** 10.1155/2014/693048

**Published:** 2014-11-24

**Authors:** Mohamed Elsayed Khallaf, Ahmed Maher Gabr, Eman Elsayed Fayed

**Affiliations:** ^1^Department of Physical Therapy for Neuromuscular Disorders and Its Surgery, Faculty of Physical Therapy, Cairo University, 7 Ahmed El-Zayat Street, Dokki, Giza, Egypt; ^2^Department of Physical Therapy for Growth and Development Disorders in Children and Its Surgery, Faculty of Physical Therapy, Cairo University, Egypt; ^3^Department of Physical Therapy, College of Applied Medical Sciences, University of Hail, Saudi Arabia

## Abstract

*Background and Purpose*. Equinovarus foot is a common sign after stroke. The aim of this study is to investigate the effect of task specific exercises, gait training, and visual biofeedback on correcting equinovarus gait among individuals with stroke. *Subjects and Methods*. Sixteen subjects with ischemic stroke were randomly assigned to two equal groups (G_1_ and G_2_). All the patients were at stage 4 of motor recovery of foot according to Chedoke-McMaster Stroke Assessment without any cognitive dysfunction. E-med pedography was used to measure contact time, as well as force underneath hind and forefoot during walking. Outcome measures were collected before randomization, one week after the last session, and four weeks later. Participants in G_1_ received task specific exercises, gait training, and visual biofeedback and a traditional physical therapy program was applied for participants in G_2_ for 8 weeks. *Results*. Significant improvement was observed among G_1_ patients (*P* ≤ 0.05) which lasts one month after therapy termination. On the other hand, there were no significant differences between measurements of the participants in G_2_. Between groups comparison also revealed a significant improvement in G_1_ with long lasting effect. *Conclusion*. The results of this study showed a positive long lasting effect of the task specific exercises, gait training, and visual biofeedback on equinovarus gait pattern among individuals with stroke.

## 1. Introduction

Gait deficits are associated with significant functional limitations. They can make it difficult for individuals after stroke to move about in their home [1]. Community access requires not only the ability to walk but also the ability to manage uneven surfaces, elevations, and curbs [2]. After stroke, sensorimotor impairments including proprioceptive deficits, muscle weakness, spasticity, and stereotyped movement interfering with normal gait are common and are associated with an increased risk of falling [2, 3]. Although hemiparetic gait has been investigated in many studies and many rehabilitation methods have been developed in order to improve motor recovery, the cause and effect relationship between impairment caused by stroke and poststroke gait pattern has yet to be fully understood [4, 5].

Hemiparetic gait pattern is characterized by being stereotyped with reduced weight bearing on the paretic lower limb (LL). Contacting the floor while the foot is flat is one of the most common stance phase kinematics disturbances. Overactivity or shortening of plantar flexor muscles at swing phase limits dorsiflexion of the ankle and causes the foot to be flat at initial contact. Increased invertors spasticity additionally allows only the lateral border of the foot to contact the floor first, giving the equinovarus foot placement [6, 7].

Previous studies have shown that the effects of muscle strengthening in seated position among poststroke people lead to inconsistent effects on gait [8, 9]. Moreover, Kim et al. state that intervention aimed at increasing strength does not result in improvements in walking [10].

Task specific training programs incorporate the practice of functional movements in a real situation with an objective to help patients to gain optimal control strategies for improving motor control [11]. In such programs, the patient is put in a situation in which the weakened muscle would normally function [12]. For task specific training, the two main approaches are found in the literature to improve gait pattern. One approach is treadmill training. The other one is intensive practice of a wide variety of functional mobility tasks. These mobility tasks can be provided through functional strengthening and endurance components [13]. Many studies concluded that having more functional ways of strengthening the muscles, such as in the context of task-specific gait training, might lead to a greater carryover effect and impact on gait [8–10]. Gait pattern has been shown to be improved in patients with stroke after a combined task-specific training program and specific strengthening exercises [14]. In the same context, functional MRI showed a positive effect of LL task specific training programs on inducing use-dependent plastic changes of brains [15, 16].

Biofeedback (BFB) is information provided from an external source, which is additional to the perception of the mover. BFB can be verbal or nonverbal. It can be provided concurrently, immediately following, or much later than the action [17]. BFB can be considered as the essence of exercise therapy for patients after stroke to compensate for the inefficient intrinsic feedback. Visual BFB from a monitor connected to load platforms was used for balance and weight shifting training in two patients after stroke. The results showed that ability to shift weight onto the affected leg improved only when training with this device and not with conventional therapy [18, 19]. Moreover, in a controlled long-term trial with stroke patients, stance symmetry, gross motor function, and activities of daily living showed a significantly greater improvement with those who received visual BFB from a monitor connected to a load platform [20]. Based on the fact that real-time BFB can facilitate a return to normal function in people following stroke [21], we sought to implement an approach using both task specific training (intensive practice of a wide variety of functional mobility tasks) and visual BFB to correct the equinovarus foot placement after stroke. In this study, we used task specific exercises, gait training, and a new method of visual BFB to test the hypothesis that patients after stroke are able to control distal spastic/weak muscles and correct equinovarus foot placement.

## 2. Subjects and Methods

**Figure 1:**
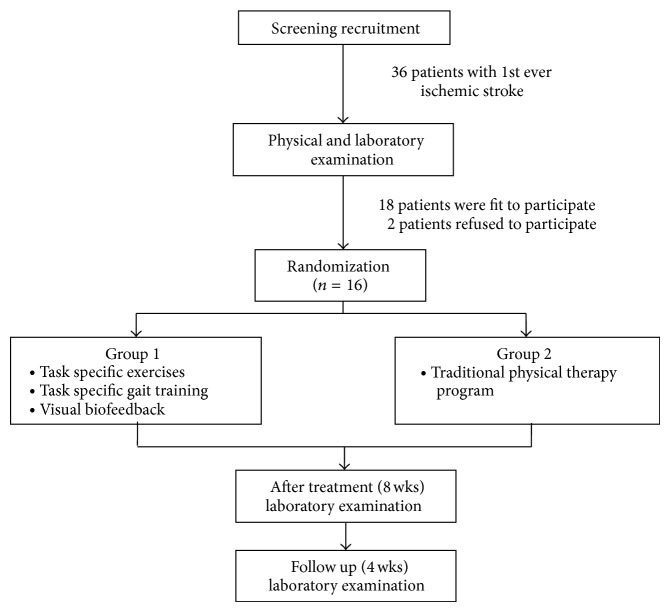
Flow of study from screening to completion of the follow-up assessment.

Sixteen subjects (4 females and 12 males) with ischemic stroke represented the sample of the study. The patients were recruited from the Physical Therapy Department, King Khalid Hospital, Hail, KSA. The study was approved by the University of Hail Local Ethical Committee. The participants were required to meet the following criteria for inclusion in the study: having history of unilateral first ever stroke causing hemiparesis with duration of illness not less than 3 months, being medically stable, having the ability to understand procedures of experiment and give study consent form, and being in at least stage 4 of motor recovery of foot according to Chedoke-McMaster Stroke Assessment with spasticity less than grade 2 by Modified Ashworth Scale (MAS). The subject should also be able to walk independently with or without assistive devices for six minutes. Patients with LL sensory impairment or cognitive, mental, and visual deficits were excluded from the study. Patients with ankle, knee contractures or who were receiving any muscle relaxant were also excluded from the study. After examining 32 patients, 18 patients were fit to participate in the study, two of them refused to participate in the study because they are living in rural areas away from the city. For treatment allocation, computer-generated random numbers were used to assign patients to two equal groups (G_1_ and G_2_). Allocation in either study or control group was concealed from the patients and researchers (Figure 1).

Before randomization and after giving study consent form, physical examination was done for all participants. It included a review of medical history, mentation assessment (using Mini Mental State Examination), neurological examination, and level of activities using an International Physical Activity Questionnaire (IPAQ). Chedoke-McMaster Stroke Assessment was used to detect the stage of foot, leg motor recovery, and the functional level (activity inventory) [22]. Spasticity was measured using the Modified Ashworth Scale (MAS).

A capacitance-based pressure platform (emed-q100, GmbH, Novel Munich, Germany) was used for detecting the pattern of foot placement. Reliability of the platform has been proved by Putti et al. [23]. The pressure platform was 700 × 403 mm, with 6080 sensors and resolution of four sensors per cm^2^ when data are collected at 100 Hz. After demonstration, participants were asked to walk barefoot across the platform at a walking speed similar to the usual. The participants were asked to focus on a rounded sticker fixed on both directions of walking at the same level to standardize gaze away from the pressure platform during measurement. As we used a long walkway, participants were asked to take four steps prior to hitting the platform and continue afterward. These procedures were repeated until five passes were obtained (five recordings of affected foot). A trial was repeated if the foot is placed near to or on the edges of the platform. The data from an average of the five steps on affected foot were used to represent the individual's dynamic foot placement. For analysis of the force and time-related measures, the foot was divided into 10 regions hindfoot, midfoot, first metatarsal, second metatarsal, third metatarsals, forth metatarsals, fifth metatarsals, big toe, second toe, and toes 3, 4, and 5. Novel's foot report (Novel, Munich, Germany) was used to provide mathematical measurement of the foot placement in addition to colored graphs simulating foot placement. These reports were created immediately after the data collection/measurement. Three measurements were done for each patient, at baseline (1st measurement), after treatment (2nd measurement), and one month after the end of the treatment (3rd measurement).

Patients in the study group (G_1_) received intensive mobility training which included manual stretching, muscle-specific progressive-resistive exercise, balance training, and walking program (50–70% age adjusted heart rate maximum) with BFB from the E-med pedography. Manual prolonged stretch technique was applied for the calf and hip adductor muscles with holding time of 30 seconds. Manual resistance exercises were followed by theraband strengthening for foot evertors, ankle dorsiflexors, knee flexors, hip extensors, hip abductors, and knee extensors. The rational for using manual resistance is to ensure that the participant is doing the intended movements without any substitutions or interference of associated reactions and being isokinetic in nature. As the participant is able to master the proposed movement, therabands were used for increasing the number of repetitions and resistance. Functional strengthening exercises were also used for evertors and hip abductors. Patients were asked to do abduction while standing against a wall by sliding the heel against the wall with weight cuffs just above the ankle. Sideway walking onto blocks was used for the same groups of muscles. An exercise like horse pawing was used for increasing knee flexors and planter flexors strength as preparation for gait training [24]. With fully extended knees, patients were also asked to raise, hold, and lower the forefoot from the floor midway between eversion and inversion while standing against wall. Knee taps to the wall and wall calf stretch exercises were practiced for stretching the calf muscles and forward pivot training. For balance training, step up and sideways onto a step, chair rise, marching, kicking a ball laterally, stops and turns while walking were used. These exercises are applied for 90 minutes 5 times a week for 8 weeks.

**Figure 2:**
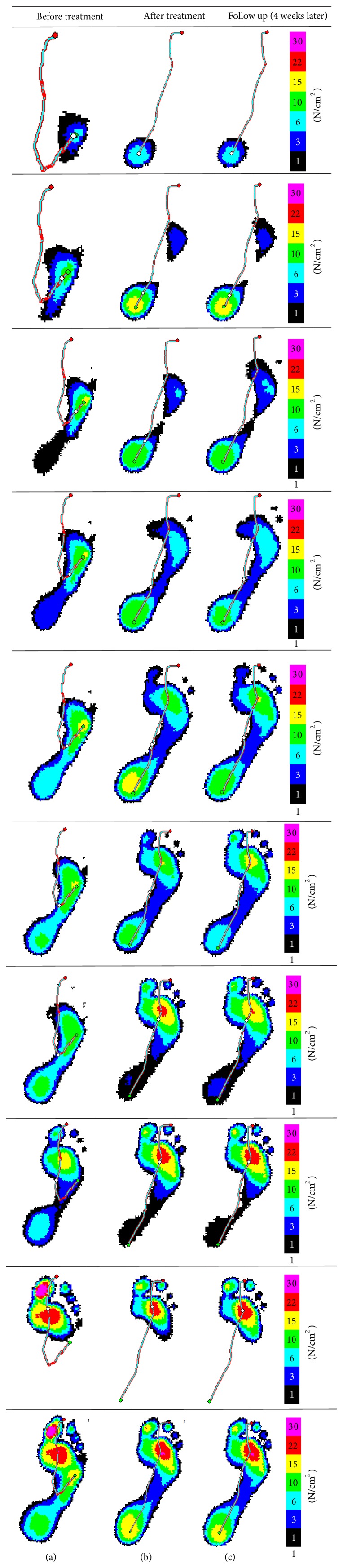
Averaged maximum pressure: (a) before treatment, (b) after 8 weeks of treatment, and (c) one month after treatment. In this figure (a) the patient started placing the foot on the floor by the lateral border of the foot as shown by the pathway of the center of pressure (rollover). In (b) and (c), the center of pressure starts at the heel and passes in the middle of the foot indicating balanced forefoot placement and appropriate timing. The last row shows the averaged maximum pressure of all frames.

Participants in G_1_ received also walking program (50–70% age adjusted heart rate maximum) with biofeedback from the pedography. During gait training, the patient was instructed to step over the E-med platform five times. Data base professional was used for foot placement analysis. The obtained results were used as a teaching material. The colors of the graphs (Figure 2) and foot rollover were the key point for the patients to understand the impairment of foot placement.

Patients allocated in the control group (G_2_) received a program of strengthening exercises for the foot evertors and ankle dorsiflexors in addition to prolonged stretching of the calf muscles. The patients also received gait training with cones between the parallel bars. Participants were given five sessions per week for 8 weeks, 50 minutes for each session. A solid ankle foot orthosis (AFO) was also used as a traditional treatment of the foot equinovarus deformity. The patients were instructed to use the AFO for at least eight hours a day. For either groups, the treatment programs were done by a neurophysiotherapists.

*Statistical Analysis.* Descriptive statistics were calculated to summarize the demographic characteristics of the sample and all outcome measures at baseline (1st measurement), postintervention (2nd measurement), and one month after intervention (3rd measurement) for each group. Demographic data was compared between groups by *t*-test (*P* ≤ 0.05). Repeated measures of ANOVA were employed to calculate, within subjects, effect of treatment programs in the study and control group at probability level equal to or less than 0.05. Between groups, effects of the applied treatment programs were compared using *t*-test with level of significance set at *P* ≤ 0.05.

## 3. Results

The characteristics of the participants are shown in Table 1. The two groups were matched for age and sex (*P* = 0.213, 1.00), body weight (*P* = 0.513), body height (*P* = 0.163), shoe size (*P* = 0.732), and time since onset of stroke (*P* = 0.442). There were nine patients with right sided and seven with left sided hemiparesis (*P* = 0.351). Hypertension and diabetes mellitus were represented in both groups with a percentage of 62.5% and 25% in group 1 and 75% and 37.5% in group 2, respectively. Fifty percent of the participants in group 2 were smokers before the onset of stroke, while 62% of people who were presented in group 1 used to smoke. Obesity and level of activity were equally represented in both groups.

Clinical examination of the participants revealed that the participants of both groups have an increase of muscle tone (spasticity) of grades 1 and 1+ MAS. According to Chedoke-McMaster Stroke Assessment, participants in both groups were in stages 4 and 5 of foot and leg motor recovery, respectively. Activity inventory was employed to assess the participants' functional level, which was 6 in both groups (Table 2).

A repeated measure of ANOVA was employed to measure, within subjects, changes after treatments. In study group (G_1_), there were significant changes observed in the maximum force and contact time in different foot areas. There was a significant increase of the maximum force (*P* = 0.001) underneath the hindfoot with significant increase (*P* = 0.001) in the contact time indicating restoration of the initial contact of the foot with the ground. For the first, second, and fifth metatarsal heads, there was a highly significant change (*P* = 0.001) after the application of BFB and motor relearning therapies, which extended to 4 weeks after the end of the treatment time. A less but significant change was observed in the contact time and maximum force of the third and fourth metatarsal heads. The contact time of the five metatarsals was significantly lower (*P* = 0.001) than the baseline measurement. In the control group, statistical analysis of the results revealed a minimal, nonsignificant change in the contact time and maximum force of the ten anatomical areas of the foot after treatment and the follow-up measurements (Tables 3 and 4).

A paired *t*-test was used for enlightening the difference in improvement between groups (Tables 3 and 4). There were nonsignificant differences among the groups in the baseline measurements of the contact time and force recorded at different regions of the foot. For the hindfoot, there was a highly significant difference in both contact time and maximum force after treatment which last to the follow-up measurement (*P* = 0.001). In the same context, significant differences were observed in the contact time (*P* = 0.001, 0.035) and maximum force (*P* = 0.004, 0.001) recorded underneath the fourth metatarsal head. Significant differences were also observed in the contact time (*P* = 0.001) and maximum force (*P* = 0.001) recorded underneath the fifth metatarsal head after treatment and follow-up measures. Less but significant differences were observed in the contact time (*P* = 0.026, 0.022) and maximum force (*P* = 0.001) recorded underneath the first metatarsal head after treatment and follow-up measures. For the second metatarsal head, there were significant differences in the contact time (*P* = 0.023, 0.024) and maximum force (*P* = 0.037, 0.008).

## 4. Discussion

We report the results of a therapeutic program that included task-specific exercises and gait training in combination with visual BFB. This study represents a novel application of a pedography for providing visual BFB during walking for correction of equinovarus foot placement after stroke. Inadequate strength and hypertonia of the muscles controlling the foot result in disturbance in force distribution among the foot regions. In this study, before treatment there was an increased force distribution under the fourth and fifth metatarsal heads which is significantly decreased after treatment. On the other hand, the lowered force under the first and second metatarsals is significantly increased after treatment. In the same context, the recorded force under the hindfoot is significantly increased after treatment. Figure 2 shows the difference between the foot placement before and after treatment. Before treatment, the center of pressure started at the area of the fourth and fifth metatarsal heads. After treatment, the center of foot pressure advancement started at the heel and midway throughout the forefoot indicating restoration of the initial contact. This also indicates that the foot is placed midway between inversion and eversion. This noticeable persistent change (as indicated by follow-up measure one month after treatment) of the foot placement can be attributed to an improvement in motor control of the paretic foot and an increased ability to do movements with greater selectivity at the ankle and foot while walking. This improvement is a result of using task-specific intensive training in association with visual BFB training which enhances the motor control. Our results are in close agreement with Perry and Rodgers et al. The authors reported that improvement in foot placement is a result of enhanced neuromuscular responsiveness in the paretic limb and using the limb in a more controlled manner [25, 26]. This is also consistent with Mizelle et al. who stated that improved motor unit activation associated with increased gait key muscle functional strength is the cause behind improved gait pattern after stroke [27]. Our findings are in general agreement with those who reported greater improvements in functional abilities, including transfers and gait, following treatment that included biofeedback/force plate training [28, 29].

The hindfoot time of contact with the floor is significantly increased in the study group. On the other hand, the forefoot time of contact with the floor is lowered. This is an indication that the early stance time is increased not only as a sequence of increased ability to control the calf muscle spasticity but also as a result of enhanced eccentric control over the forward pivoting of the leg on the foot. Increasing the hindfoot contact time means restoration of the initial contact, loading response, and midstance subphases. This is the opposite of the hemiparetic gait pattern with equinovarus foot [30].

The effects of the treatment still persisted at follow-up, indicating that the process of motor relearning has been completed with improved progression and timing of movement. This can be attributed to the effect of repeated practice of a walking with extrinsic BFB. We employed the visual BFB as a part of functional training to provide specific information that can be used to adapt the next attempt of walking over sixty hours of intensive training. This is consistent with Geiger et al. and Van Peppen et al., who stated that motor learning and recovery indicate that intervention should be meaningful, task-specific, and tailored to the person's capacity and interests and provide sufficient repetition and challenge to induce training effects [30, 31].

Utilization of the pedography as a source of foot placement biofeedback is considered a good shortcut for the patient to understand how he/she places the foot on the floor. The BFB applied in this study is different from other forms of visual BFB. It is task specific, provides accurate and reliable scanning of the foot placement during walking, and provides easy, understandable information for the patient to correct his/her foot placement. It is also unlike the electromyographic BFB, which depends on the electrical activities of the muscles during static situations in most of its applications. For these reasons, BFB is of great importance for motor relearning [32]. This is consistent with De Weerdt et al. and Sackley et al., who used load platforms linked to a computer to train stroke patients' balance and weight shift with visual BFB from a monitor. The experiment showed that there is improvement in the ability to shift weight onto the affected leg only when training with this device and not with conventional therapy or another feedback training of the arm [18–20].

In this study, we have a limitation of a small sample size; nevertheless, we were able to show the benefits of using task specific biofeedback and exercises for correcting foot placement after stroke. This trend of changes that we observed warrants further study with a larger sample size and wearable technology to enable outdoor activities and a more discerning statistical analysis.

## 5. Conclusions

This study offered a real functional situation for training, which has a positive influence on gait after stroke. Pedographic visual BFB in combination with task specific training can be used effectively for correction of equinovarus gait among stroke patients.

## Figures and Tables

**Table 1:** Subject characteristics, values presented as mean (SD) and percentages.

	Group 1 *n* = 8	Group 2 *n* = 8	*P*
Age	40.38 ± 2.67	41.25 ± 3.11	0.213
Sex (male/female)	6/2	6/2	1.00
Weight (Kg)	83.13 ± 4.32	82.13 ± 3.27	0.513
Height (cm)	175.75 ± 11.93	171.88 ± 10.22	0.163
Shoe size	41.75 ± 2.12	41.38 ± 2.62	0.732
Paretic right side	5	4	0.351
Duration of illness (m)	9.88 ± 2.80	10.00 ± 2.39	0.442
Hypertensive	62.5%	75%	—
Diabetic	25%	37.5%	—
Smokers	62.5%	50%	—
Obese	25%	25%	—
Level of activity	Low	Low	—

**Table 2:** Muscle tone assessment (percentage) and recovery and functional level.

Groups	Modified Ashwarth's scale		Chedoke-McMaster Stroke Assessment		
	Grade 1	Grade 1+	Foot stage	Leg stage	Activity inventory (ambulation)
Group 1 (*n* = 8)	25%	75%	4	5	6
Group 2 (*n* = 8)	37.5%	62.5%	4	5	6

**Table 3:** Mean scores of time of contact (percentage average rollover period) for the study and control groups and the associated *P* values for test of differences.

		Group 1	Group 2	Between groups		Within subject effect			
Foot area	Measures			effect		Group 1		Group 2	
		Mean ± SD	Mean ± SD	*T*	*P*	*F*	*P*	*F*	*P*
Hindfoot	1st	53.62 ± 3.49	50.38 ± 1.16	2.15	0.069	77.31	0.001^*^	2.109	0.163
	2nd	67.03 ± 1.49	51.84 ± 3.63	11.02	0.001^*^				
	3rd	62.69 ± 1.98	49.09 ± 3.14	12.25	0.001^*^				

First metatarsal head	1st	92.29 ± 1.09	90.08 ± 2.72	2.14	0.096	95.68	0.001^*^	1.950	0.194
	2nd	82.84 ± 1.37	88.19 ± 4.77	2.82	0.026^*^				
	3rd	86.38 ± 1.45	89.89 ± 3.32	2.92	0.022^*^				

Second metatarsal head	1st	94.41 ± 2.72	90.88 ± 3.86	1.87	0.103	79.13	0.001^*^	0.628	0.523
	2nd	85.36 ± 1.15	89.92 ± 4.75	2.58	0.037^*^				
	3rd	84.63 ± 1.09	88.88 ± 2.49	3.66	0.008^*^				

Third metatarsal head	1st	94.46 ± 2.34	91.85 ± 5.30	1.24	0.254	5.61	0.016^*^	3.496	0.060
	2nd	91.61 ± 2.83	86.48 ± 5.21	2.72	0.030^*^				
	3rd	91.09 ± 1.06	85.84 ± 3.90	4.02	0.005^*^				

Forth metatarsal head	1st	92.48 ± 1.23	90.14 ± 5.46	1.21	0.263^*^	10.99	0.001^*^	0.300	0.731
	2nd	81.20 ± 1.29	90.95 ± 4.62	5.32	0.001^*^				
	3rd	84.83 ± 2.82	89.74 ± 4.75	2.62	0.035^*^				

Fifth metatarsal head	1st	86.09 ± 2.82	85.95 ± 2.07	0.11	0.913	38.57	0.001^*^	0.393	0.679
	2nd	77.32 ± 1.47	84.57 ± 3.90	5.61	0.001^*^				
	3rd	77.66 ± 1.45	85.34 ± 3.23	5.86	0.001^*^				

^*^ *P* is significant at *P* < 0.05.

**Table 4:** Mean scores of maximum force (N/cm^2^) for the study and control groups and the associated *P* values for test of differences.

		Group 1	Group 2	Between groups		Within group effect			
Foot area	Measures			effect		Group 1		Group 2	
		Mean ± SD	Mean ± SD	*T*	*P*	*F*	*P*	*F*	*P*
Hindfoot	1st	124.13 ± 6.47	122.14 ± 5.05	0.759	0.472	87.82	0.001^*^	0.263	0.772
	2nd	323.47 ± 24.7	125.22 ± 13.52	30.568	0.001^*^				
	3rd	313.72 ± 23.3	122.55 ± 14.16	32.942	0.001^*^				

First metatarsal head	1st	126.37 ± 9.2	128.49 ± 5.17	0.851	0.423	64.21	0.001^*^	0.532	0.599
	2nd	95.32 ± 3.45	132.32 ± 5.32	23.074	0.001^*^				
	3rd	97.46 ± 4.03	129.96 ± 8.97	10.370	0.001^*^				

Second metatarsal head	1st	173.7 ± 10.6	168.95 ± 7.13	1.305	0.233	18.94	0.001^*^	2.180	0.438
	2nd	149.3 ± 5.31	162.02 ± 10.89	2.906	0.023^*^				
	3rd	147.9 ± 10.3	157.36 ± 8.16	2.864	0.024^*^				

Third metatarsal head	1st	194.65 ± 2.9	190.27 ± 4.66	2.150	0.690	26.81	0.001^*^	1.337	0.294
	2nd	203.4 ± 3.79	195.07 ± 7.33	3.572	0.009^*^				
	3rd	200.1 ± 1.99	193.74 ± 7.78	2.404	0.047^*^				

Forth metatarsal head	1st	144.2 ± 9.65	141.19 ± 7.02	1.228	0.259	28.73	0.001^*^	0.512	0.610
	2nd	120.77 ± 5.3	142.77 ± 8.84	4.303	0.004^*^				
	3rd	122.61 ± 4.4	145.24 ± 10.52	6.446	0.001^*^				

Fifth metatarsal head	1st	43.64 ± 4.9	47.52 ± 3.16	0.694	0.510	28.75	0.001^*^	1.617	0.233
	2nd	61.77 ± 3.1	51.52 ± 5.19	6.469	0.001^*^				
	3rd	56.67 ± 3.1	52.42 ± 6.79	3.434	0.001^*^				

^*^ *P* is significant at *P* < 0.05.
